# Rapid detection of terbinafine resistance in *Trichophyton* species by Amplified refractory mutation system-polymerase chain reaction

**DOI:** 10.1038/s41598-020-58187-0

**Published:** 2020-01-28

**Authors:** Shamanth A. Shankarnarayan, Dipika Shaw, Arunima Sharma, Arunaloke Chakrabarti, Sunil Dogra, Muthu Sendhil Kumaran, Harsimran Kaur, Anup Ghosh, Shivaprakash M. Rudramurthy

**Affiliations:** 1 Department of Medical Microbiology, Postgraduate Institute of Medical Education and Research, Chandigarh, 160012 India; 2 Department of Dermatology, Venerology & Leprology, Postgraduate Institute of Medical Education and Research, Chandigarh, 160012 India

**Keywords:** Fungal genetics, Molecular medicine

## Abstract

Dermatophytosis has gained interest in India due to rise in terbinafine resistance and difficulty in management of recalcitrant disease. The terbinafine resistance in dermatophytes is attributed to single nucleotide polymorphisms (SNPs) in squalene epoxidase (*SE*) gene. We evaluated the utility of amplified refractory mutation system polymerase chain reaction (ARMS PCR) for detection of previously reported point mutations, including a mutation C1191A in the *SE* gene in *Trichophyton* species. ARMS PCR was standardized using nine non-wild type isolates and two wild type isolates of *Trichophyton* species. Study included 214 patients with dermatophyte infection from March through December 2017. Antifungal susceptibility testing of isolated dermatophytes was performed according to CLSI-M38A2 guidelines. Among dermatophytes isolated in 68.2% (146/214) patients, *Trichophyton* species were predominant (66.4%). High (>2 mg/L, cut off) minimum inhibitory concentrations to terbinafine were noted in 15 (15.4%) *Trichophyton mentagrophytes* complex isolates. A complete agreement was noted between ARMS PCR assay and DNA sequencing. C to A transversion was responsible for amino acid substitution in 397^th^ position of *SE* gene in terbinafine resistant isolates. Thus, the ARMS PCR assay is a simple and reliable method to detect terbinafine-resistant *Trichophyton* isolates.

## Introduction

Dermatophytosis, the commonest cutaneous fungal infection has drawn attention in recent years in India due to a change in the clinical profile and an upsurge in the number of chronic/recurrent/recalcitrant dermatophytosis cases^1^. Extensive infection, atypical presentation, poor response or non-responsiveness to the recommended antifungals has made dermatologists perplexed about management and control of the disease. Although antifungal resistance has been proposed as one of the causes for treatment unresponsiveness, there may be other reasons as well, as treatment failure cases outnumber the number of resistant cases. The current guidelines for management of the chronic/recurrent/recalcitrant cases recommend accurate dermatophyte identification and antifungal susceptibility testing^2^. Terbinafine being a fungicidal drug, is recommended for systemic therapy. However, recent reports from the subcontinent has revealed an emerging trend of terbinafine resistance in *Trichophyton* species. A recent study from India demonstrated low cure rates of 2% at 2 weeks duration and 30.6% at 4 weeks in patients treated with terbinafine^3^. Terbinafine resistance in *Trichophyton mentagrophytes* complex and *Trichophyton rubrum* has been ascribed to point mutation in the squalene epoxidase (*SE*) gene, the gene essential for the ergosterol biosynthesis^4–6^.

With the advent of the molecular techniques, identification of the dermatophytes to the species level can now be achieved rapidly and accurately. However, antifungal susceptibility testing (AFST) of the dermatophytes is still not well standardized. The current recommendations of Clinical and Laboratory Standard Institute (CLSI) lack consistency in correlating *in vitro* minimum inhibitory concentration (MIC) data with the clinical outcome. However, with the increasing burden of terbinafine resistance among *Trichophyton* species, it is essential to identify the resistant isolates early for appropriate management.

The allylamine resistance mechanism among *Trichophyton* species is attributed to single nucleotide polymorphisms (SNPs) in the *SE* gene. Point mutations in the *SE* gene lead to the amino acid substitution in one of the four positions (﻿Leu393, Phe397, Phe415, His440), which correspond to elevated MICs to terbinafine *in vitro*^4^. On the contrary, novel missense mutations leading to H440Y/F484Y and I121M/V237I substitutions in *SE* gene were reported in isolates with low level resistance^7^. Multiple molecular methods have been developed and evaluated for the effective detection of SNPs of interest in other fungi^8^. Among those methods’ DNA sequencing is the best genotyping tool, but it is expensive, time consuming and not available in majority of the routine diagnostic laboratories.

Amplification refractory mutation system polymerase chain reaction (ARMS PCR), also known as allele specific polymerase chain reaction, is an efficient technique for identification of SNPs and deletion mutations^9,10^. Tetra primer ARMS PCR has been used for the detection of inherent and acquired mutations^11,12^. ARMS PCR technique uses a set of allele-specific PCR primers that allows amplification of alleles carrying mutation and do not amplify the wild type allele. The PCR generates two amplicons with one large sized product serving as an internal control and the other shorter amplicon as the allele-specific product. The positive amplification of this allele-specific PCR product is diagnostic of the target allele. In the present study, we evaluated the utility of ARMS PCR for the detection of SNPs in the *SE* gene responsible for terbinafine resistance from the genomic DNA isolated from *Trichophyton* species.

## Results

### Patient details and identification

Dermatophytes were isolated from the skin scrapings of 146/214 (68.2%) patients; *T. mentagrophytes* complex from 97 (66.4%) patients; *T. rubrum* from 41 (28.0%) patients and other dermatophytes in 8 (5.6%) patients. Tinea corporis (64, 43.8%) was the predominant clinical form followed by tinea cruris (31,21.2%), tinea pedis (n = 4, 2.7%), tinea faciei (3, 2.1%), tinea mannum (2, 1.4%) and tinea barbae (1, 0.7%). Multiple sites were involved in 41 (28.1%) patients. AFST revealed rise in MICs (2–16 mg/L) to terbinafine in 15 *T. mentagrophytes* complex isolates. Details on prior exposure to antifungal agents and relapse cases are presented in Table 1. The sequencing of ITS portion of ribosomal DNA of the 15 isolates showed similarity with the neotype *T. mentagrophytes* (IHEM 4268NT, 595/597 base pairs) and neotype *T. interdigitale* (CBS428.63NT, 593/597 base pairs). The sequencing of 28 S region (accession Nos. MK967531 to MK96755) as well as partial β-tubulin gene region (accession Nos. MK982906-MK982925) could not conclusively differentiate *T. mentagrophytes* and *T. interdigitale*.

**Table 1:** Salient features of dermatophytosis due to terbinafine resistant *Trichophyton* species.

Patient ID	Organism	NCCPF No./ITS accession No/*SE* gene accession No.	Antifungal exposure	Relapse	MIC(mg/L) Terbinafine	Amino acid substitution	Nucleotide substitution
27/17	*T. mentagrophytes* complex	800052/MH517546/MH618757	Terbinafine	No	2	F397L	^1189^TTC → CTC
50/17	*T. mentagrophytes* complex	800053/MH517547/MH618758	Terbinafine	No	2	F397L	^1189^TTC → CTC
60a/17	*T. mentagrophytes* complex	800060/MH517548/MH618759	No	Yes	2	F397L	^1189^TTC → CTC
60b/17	*T. mentagrophytes* complex	800061/MH517549/MH618760	Terbinafine	Yes	2	F397L	^1189^TTC → CTC
69/17	*T. mentagrophytes* complex	800054/MH517550/MH618761	No	Yes	8	F397L	^1189^TTC → CTC
91/17	*T. mentagrophytes* complex	800066/MH517551/MH618762	Terbinafine	No	8	F397L	^1189^TTC → TTA
100/17	*T. mentagrophytes* complex	800059/MH517552/MH618763	No	No	8	F397L	^1189^TTC → CTC
110b/17	*T. mentagrophytes* complex	800065/MH517553/MH618764	Terbinafine	Yes	16	F397L	^1189^TTC → TTA
134b/17	*T. mentagrophytes* complex	800057/MH517554/MH618765	Luliconazole	No	8	F397L	^1189^TTC → CTC
134c/17	*T. mentagrophytes* complex	800058/MH517555/MH618766	Terbinafine	No	8	F397L	^1189^TTC → CTC
135/17	*T. mentagrophytes* complex	800056/MH517556/MH618767	No	No	4	F397L	^1189^TTC → CTC
54/17	*T. mentagrophytes* complex	800063/MH517557/MH618768	No	No	2	F397L	^1189^TTC → TTA
138/17	*T. mentagrophytes* complex	800055/MH517558/MH618769	No	No	16	F397L	^1189^TTC → CTC
176/17	*T. mentagrophytes* complex	800064/MH517559/MH618770	No	Yes	8	F397L	^1189^TTC → TTA
200/17	*T. mentagrophytes* complex	800062/MH517560/MH618771	No	Yes	8	F397L	^1189^TTC → TTA

### Antifungal susceptibility testing and *SE* gene sequencing

The terbinafine-resistant *T. mentagrophytes* complex isolates obtained in this study had MIC range between 2 to 16 mg/L. There was no difference in the MIC of the sequential isolates from two patients isolated at two different time points. Analysis of the *SE* gene showed two important mutations in the coding region responsible for the changes in the amino acid sequence. In addition to the previously described point mutation at T1189C in *SE* gene, we also observed mutation at 1191^th^ position leading to same amino acid substitution at 397^th^ position in the *SE* gene of *T. mentagrophytes* complex. Among 15 *T. mentagrophytes* complex isolates with higher MICs to terbinafine, 10 isolates had T1189C mutation, whereas 5 isolates had C1191A mutation. The *SE* gene sequence of clinical isolates with lower MICs (NCCPF 800035, 800036, 800038) to terbinafine exhibited wild type genotype.

### Standardization and validation of ARMS PCR assay

**Figure 1:**
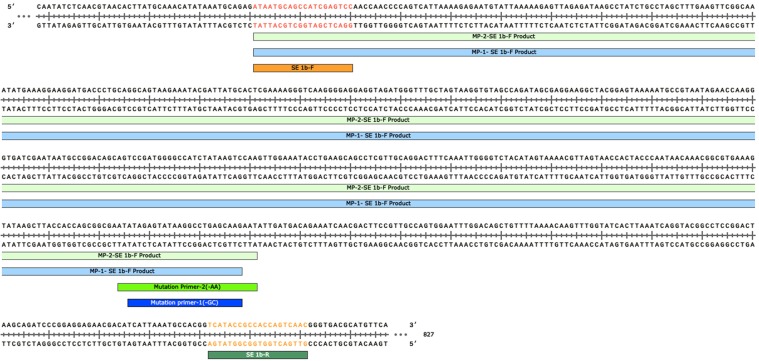
Primer sequences and positions used in the ARMS PCR assay for wild type and non-wild type genotyping of *Trichophyton* species. During the amplification reaction a 611 bp region of *SE* gene was amplified with common primers that operated as internal control for the quality of the PCR amplification. The mutant specific amplification obtained 449 bp and 451 bp PCR products specific for T1189C and C1191A mutant types respectively.

**Figure 2:**
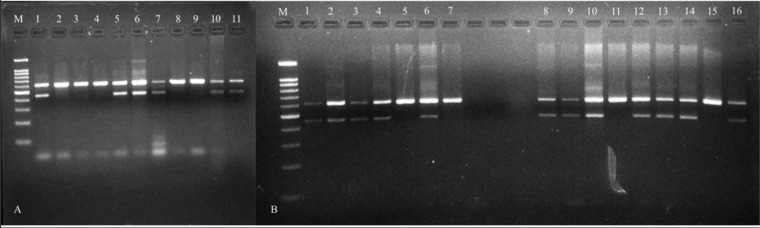
(**A**) Standardization of modified ARMS PCR assay from the genomic DNA of *Trichophyton mentagrophytes* complex and *Trichophyton rubrum*; Lane 1 = *Trichophyton rubrum* (NCCPF 900041) with higher MICs to terbinafine having T1189C mutation; Lane 2, 3, 4 = *Trichophyton mentagrophytes* complex (NCCPF 800035, 800036, 800038) with low MICs to terbinafine. Lane 5, 6 = *Trichophyton mentagrophytes* complex (NCCPF 800022, 800023) with higher MIC’s to terbinafine having T1189C mutation; Lane 7,10,11 *Trichophyton mentagrophytes* complex (NCCPF 800062, 800063, 800064) with higher MICs to terbinafine having C1191A mutation; Lane 8, 9 = *Trichophyton mentagrophytes* complex (NCCPF 800035, 800036) with low MICs to terbinafine. Lane 1–6 were amplified using mutation primer-1 and lane 7–11 amplified using mutation primer-2. (M = 100 base pair ladder). (**B**) Validation of the modified ARMS PCR assay against genomic DNA of representative *T. mentagrophytes* complex with higher MICs to terbinafine. Lane 1, 2, 3, 4, 6 = *Trichophyton mentagrophytes* complex (NCCPF 800062, 800063, 800064, 800065, 800066) with high terbinafine MIC’s and C1191A mutation; lane 5, 7 = *Trichophyton mentagrophytes* complex (NCCPF 800043, 800044) with low MICs to terbinafine; Lane 8, 9, 10, 12, 13, 14, 16 = *Trichophyton mentagrophytes* complex (NCCPF 800055, 800056, 800052, 800053, 800060, 800061, 800057) with T1189C mutation; Lane 11, 15, = *Trichophyton mentagrophytes* complex (NCCPF 800043, 800044) with low terbinafine MICs. Lane 1–7 were amplified using mutation primer-2 and Lane 8–16 amplified with mutation primer-1. (M = 100 base pair ladder).

A simple and reliable modified ARMS PCR assay was developed for the rapid detection of the most common mutations associated with the terbinafine resistance in *SE* gene of *T. mentagrophytes* complex and *T. rubrum*. The assay was evaluated against previously reported mutations and the mutation identified in this study. Using the designed primers, an amplicon of 611 base pair (bp) was amplified from the internal control primers in both mutant and wild type strains. In addition, the mutated isolates produced a second amplicon of 449 bp with mutation primer (MP) -1 and 451 bp with MP-2 primer (Figs. 1 and 2A). Reproducibility of the assay was also tested in three independent PCR reaction on three different days by different personnel. For each tested reaction one positive and negative control were included (data not shown). We observed 100% reproducibility with each test. We also evaluated the analytical sensitivity of the modified ARMS PCR assay by diluting the template DNA of four samples. After standardizing the modified ARMS PCR protocol, remaining *Trichophyton* isolates with higher MICs and lower MICs to terbinafine were included in the assay (Fig. 2B). While validating the assay, both the primers (MP-1 and MP-2) were incorporated in the PCR master mix and used for a total of 12-*T. mentagrophytes* complex isolates with higher terbinafine MICs and 10 -*T. mentagrophytes* complex isolates with lower MICs to terbinafine. The isolates harbouring mutation in the *SE* gene and the wild type isolates were successfully differentiated by the ARMS PCR assay. Post-validation, sequencing of *SE* gene was performed, which showed 100% agreement between ARMS PCR assay and DNA sequencing. Further, in the view of several inherent inhibitors in the simple PCR techniques, the samples were blindly tested independently by two other laboratory experts. These results were consistent with our earlier findings. The average time taken by the modified ARMS PCR assay to detect the terbinafine resistance after obtaining pure culture of *Trichophyton* species was approximately 24–32 hours in comparison to approximately 5–10 days taken by the susceptibility testing followed by sequencing.

## Discussion

In addition to the increased prevalence of dermatophytosis over the past 4–5 years across India, a rise in recurrent (dermatophytosis that runs a protracted course with episodes of remission and exacerbation) and chronic dermatophytosis cases has also been observed^13^. Antifungal resistance in dermatophytes is also emerging rapidly, especially to allylamines. In the present study, a rapid, simple and reliable ARMS PCR assay was developed for screening of mutations in *SE* gene conferring resistance to terbinafine. The study also reports the presence of a novel mutation (C1191A) among *T. mentagrophytes* complex isolates responsible for causing dermatophytosis in India^6,14^ These new mutant isolates harbour mutation at the 1191^th^ position leading to change in amino acid from phenylalanine to leucine at 397^th^ position. Other than the mutation identified in this study, two studies from India have reported on amino acid substitution at 397^th^ position^5,6^. In both the reports, ^1189^CTC codon encoding leucine was responsible for amino acid substitution. However, in the present study, transversion from cytosine to adenine at third base of the codon (^1189^TTA) was observed, leading to the amino acid substitution. Due to redundancy in the genetic code, both CTC and TTA codes for leucine. Previous reports emphasized that the mutation in this region modify the protein structure leading to interference in binding of the terbinafine to the target enzymes and decreased susceptibility in *Trichophyton* species^4,6,15^. In the present study, the earlier reported mutation responsible for amino acid substitution at 393^rd^ position was not detected in any terbinafine-resistant *T. mentagrophytes* complex isolates^5^. Till now 10 missense mutations (A1179T, T1178C, C1191A, T1189C, T1189A, T1189G, T1305A, T1305G, T1306C, C1380T) are attributed for amino acid substitution at four positions (Leu393, Phe397, Phe415, His440) of SE protein and resistance to terbinafine^4,14,16^. In a recent study, Saunte *et al*., identified L393S, H440Y/F484Y and I121M/V237I substitutions responsible for low to moderate terbinafine resistance^7^.

As majority of the reports highlighted the role of missense mutations responsible for allylamine resistance in dermatophytes, we developed ARMS PCR assay for rapid detection of SNPs. The SNPs can be detected by high end techniques like MLPA assay, MALDI TOF MS spectrophotometry, real time PCR assays, probe-based assays and direct DNA sequencing. But these techniques are either not available in majority of routine laboratories or not feasible because of the complexity and high cost associated with these methods. For diagnostic purposes, a fast, reliable and cheaper tests with reasonable specificity are required. In the present study, we standardized and evaluated ARMS PCR assay for genotyping terbinafine-resistant *T. mentagrophytes* complex and *T. rubrum* isolates harboring mutation. The ARMS PCR assay could easily differentiate wild type isolates from non-wild type isolates with a consensus gene specific internal control^17^.

Internal control used in this assay is gene specific, which serve as template for the subsequent amplification. This would help in amplification even in condition of low quantity of template DNA. Multiplexing primers for the detection of various reported mutations in the *SE* gene may further increase the sensitivity of detecting terbinafine-resistant *Trichophyton* species. This study could serve as a prelude to diagnosis of terbinafine resistant cases directly from the skin scrapings of patients with dermatophytosis. Further application of this technique directly to the clinical samples with appropriate controls (to check for PCR inhibitors) could reduce the turn-around time in the detection of resistant species, which needs further evaluation. The ARMS PCR assay is a rapid reliable technique compared to other currently employed methods for the detection of terbinafine resistance. This technique bypasses AFST and sequencing of *SE* gene and reduces the cost and time. The results of ARMS PCR will help the dermatologist to decide appropriate antifungal therapy for management of dermatophytosis. The only drawback of the test is that it could not delineate two bands in agarose gel, as the difference between two non-wild type genotypes were only two nucleotides. However, as both the mutations are associated with resistant phenotype, the inability to differentiate these mutations becomes insignificant and may not affect the management.

In conclusion, the ARMS PCR assay developed in this study is a simple, reliable and can be easily be employed in routine laboratories for detection of terbinafine-resistant isolates. This test also has potential to detect terbinafine resistance in dermatophytes directly in clinical samples.

## Material and Methods

### Clinical and microbiological characteristics of *Trichophyton* isolates

The work was approved by the Institutional Ethical Committee of Postgraduate Institute of Medical Education and Research, Chandigarh, India (Ethical approval number- IEC-05/2017–649). Informed consent was obtained from the patients before collecting clinical samples. All methods used in this study were performed in accordance with the relevant guidelines and regulations^18,19^.

A total of 214 patients with suspected dermatophyte infection were included in the study during March 2017 to December 2017. Skin scrapping was collected from these patients according to standard procedure and subjected to direct microscopic examination on 10% potassium hydroxide and calcofluor wet mount. Culture was performed on Sabouraud dextrose agar (HiMedia, Mumbai, India) containing chloramphenicol and cycloheximide and incubated at 28 °C up to four weeks. Any growth observed was presumptively identified on the basis of phenotypic characters and confirmed by DNA sequencing^6^. Additionally, six isolates of *Trichophyton* species (*T. mentagrophytes* complex, n = 4; and *T. rubrum*; n = 2) isolated during our earlier study and having high MIC to terbinafine along with point mutation (T1189C) in the *SE* gene were included in this study as control^6^.

### Antifungal susceptibility testing

AFST was performed for all the *Trichophyton* species against terbinafine (Sigma Aldrich) according to micro-broth dilution technique as per Clinical Laboratory Standards Institute (CLSI) M38 A2 protocol with minor modifications. Approximately 10^6^ CFU/ml conidia were harvested and counted microscopically by haemocytometer. The suspension was then diluted to 1:100 according to primary concentration. Double the final concentration (10^3^ CFU/ml) of the conidia was adjusted before adding to the drug plates. The initial inoculum corresponded to 65–70% transmittance at 530 nm in spectrophotometer^6,20^. The inoculated plates were incubated at 28 °C for a minimum of 96 hours before reading was taken. The test was read visually with endpoint as 100% growth inhibition compared to growth control. To check the purity of the inoculated plates, 10 µl of the growth from the growth control well was inoculated onto Sabouraud dextrose agar. *Candida parapsilosis* (ATCC 22019), *Candida krusei* (ATCC 6258) and *Aspergillus flavus* (ATCC 204304) were included as quality control strains.

### Sequencing of ITS region and *SE* gene

The genomic DNA of the isolates were extracted by the phenol-chloroform-isoamyl alcohol method^21^. For molecular identification, amplification and sequencing of the complete ITS region was performed using universal primer pair ITS4 and ITS5 (ITS 4–5′ -TCCTCCGCTTATTGATATGC- 3′and ITS 5–5′-GGAAGTAAAAGTCGTAACAAGG-3′). The 28 s region of rDNA and beta tubulin gene were also sequenced. To identify the mutations responsible for the terbinafine resistance in the *SE* gene, complete gene was sequenced applying primer walking technique and primer pairs SE1aF-5′ CAGAGATAATGCAGCCATCG 3′; SE1aR-5′ CCGGATTGATGTTCCTAGGT 3′; SE2aF-5′ CCACCAGCGGCGAATATAGA 3′; SE2aR-5′ AGTCCAGTGCCAGACTGATG 3′; SE3aF- 5′ AGTCTGGCACTGGACTCCAA 3′; and SE3aR-5′ ATGATGCAGCGACGGTGACA 3′ (Sigma) as described earlier^6^. Sequencing PCR was performed for both strands using the above-mentioned primers and BigDye Terminator Cycle sequencing kit version 3.1 (Applied Biosystems, Foster City, CA). Amplicons were purified and analysed on an ABI 3130 genetic analyser (Applied Biosystems). Consensus sequence was generated using forward and reverse sequences from ITS primers and *SE* primers in Bionumerics software v 7.6 (Applied Maths, Ghent, Belgium). The sequences were compared with the GenBank DNA database using the BLAST tool, the ISHAM ITS database, and the CBS database (https://blast.ncbi.nlm.nih.gov, http://its.mycologylab.org/ BioloMICSSequences.aspx, and http://www.westerdijkinstitute.nl/Collections/BioloMICSSequences.aspx)^6^.

### Primer design for modified ARMS PCR

The primers for the ARMS PCR were designed according to previously described method using Snap Gene Viewer software (GSL Biotech, Chicago, IL). The NCBI ′BLAST′ program (http://www. ncbi.nlm.nih.gov/blast) was used to check the specificity of the primers. To increase the specificity of the reaction, a mismatch was introduced at −2 position of the 3′ end of each of the mutation specific primers^10,22^. The primers used for the ARMS PCR were SE 1bF- 5′ ATAATGCAGCCATCGAGTCC 3′and SE 1bR-5′GTTGACTGGTGGCGGTATGA 3′ as internal control. Mutation specific primer for T1189C is MP -1- 5′ CTTGCTCAGGCCTTATACTCTATGC 3′ whereas for C1191A is MP - 2- 5′ ATTCTTGCTCAGGCCTTATACTCTATATAA 3′ (Fig. 1)

### Standardization and validation of ARMS PCR assay

Terbinafine-resistant isolates of *T. mentagrophytes* complex (*n* = 2; NCCPF 800022, 800023), *T. rubrum* (NCCPF 900041) with known mutation in the *SE* gene (T1189C transition) preserved in our culture collection, and terbinafine-resistant *T. mentagrophytes* complex (*n* = 3; NCCPF 800062, 800063, 80064) isolates from the present study with C1191A transversion in *SE* gene were used for standardization. Terbinafine-susceptible *T. mentagrophytes* complex (*n* = 3; NCCPF 800035, 800036, 800038) without any mutation in the *SE* gene were included as control. The reaction was set up in a total volume of 20 μl containing approximately 100 ng DNA, 1x PCR buffer, 2.5 mM MgCl_2_, 0.2 mM dNTPs, 0.05 and 0.5 pMol of internal control primers and mutation specific primers respectively, and 1 U of Taq polymerase (GeNei, Bengaluru). The PCR program on the thermal cycler (Eppendorf, Hamburg, Germany) was as follows: an initial denaturation step at 94 °C for 10 min, followed by 35 cycles of 1 min at 94 °C, 1 min at annealing temperature 58 °C, 1 min at 72 °C, and a final extension step of 10 min at 72 °C. The PCR product (10 μl) was electrophoresed on 3% agarose gel at 100 V and 400 mAmp for 45 min. The fragments were visualized under UV light in gel documentation system. To check the specificity of the primers, we performed the PCR with the DNA of an isolate with C1191A mutation and primer designed to detect T1189C mutation and also with DNA of an isolate with T1189C mutation and primer designed to detect C1191A mutation. To validate the ARMS PCR assay, 12 *T. mentagrophytes* complex isolates (includes 3 isolates NCCPF 800062, 800063, 800064 used for standardization) with higher terbinafine MICs (2 to 16 mg/L) and 10 isolates with lower terbinafine MICs (≤0.125 mg/L) were used. Post-validation, the *SE* gene was sequenced to check for the agreement between mutated isolates and ARMS PCR assay results.

## Data Availability

The data set of ITS sequences are deposited in the GenBank database with the accession number MH517546-MH517560. The *SE* gene sequences are deposited with the accession number-MH618757-MH618771. The datasets generated during and/or analysed during the current study are available from the corresponding author on reasonable request.
